# Implementation of developmental care in routine NICU practice and early clinical outcomes in preterm infants

**DOI:** 10.3389/fped.2026.1794146

**Published:** 2026-03-18

**Authors:** Halyna Pavlyshyn, Iryna Sarapuk

**Affiliations:** Department of Pediatrics No.2, I. Horbachevsky Ternopil National Medical University, Ternopil, Ukraine

**Keywords:** breastfeeding, developmental care, neonatal intensive care unit, neonatal morbidity, postnatal growth, preterm infants, skin-to-skin contact

## Abstract

**Introduction:**

Despite improved survival of preterm infants, neonatal complications and long-term morbidity remain high, highlighting the need for optimized care in neonatal intensive care units. Developmental care aims to reduce stress and better align the extrauterine environment with intrauterine conditions, and has been associated with improved short- and long-term outcomes in preterm infants. In Ukraine, its implementation is limited, and evidence on its association with clinical outcomes in routine neonatal practice is lacking. The aim of this study was to evaluate the association between the implementation of developmental care elements, including skin-to-skin contact, and early clinical outcomes in preterm infants.

**Materials and methods:**

This was a single-center, non-randomized observational before-after cohort study. The study compared outcomes of preterm infants before and after the implementation of developmental care as part of routine clinical practice, using a retrospective control group. Two groups were included: 91 infants receiving developmental care (19 extremely and 72 very preterm infants) and 119 infants receiving standard care (21 extremely and 98 very preterm infants).

**Results:**

Developmental care was associated with lower rates of late-onset sepsis in extremely (42.1% vs. 76.2%, *p* *=* 0.049; OR = 0.23) and very preterm infants (11.1% vs. 22.4%, *p* = 0.041; OR = 0.37), lower rates of intraventricular hemorrhage in very preterm infants (13.9% vs. 30.6%, *p* = 0.008; OR = 0.37), and lower stage III retinopathy of prematurity (ROP) in extremely preterm infants (26.3% vs. 61.9%, *p* = 0.025; OR = 0.22), compared with standard care. Infants receiving developmental care had shorter length of hospital stay (*p* = 0.037 and *p* < 0.001 for extremely and very preterm infants, respectively), shorter duration of mechanical ventilation in very preterm infants (*p* = 0.045), lower risk of postnatal growth failure (26.3% vs. 57.1%, *p* = 0.048; OR = 0.20 in extremely preterm; 9.7% vs. 37.8%, *p* < 0.001; OR = 0.18 in very preterm). Developmental care with skin-to-skin contact was associated with twice the rate of prolonged breastfeeding in very preterm infants (*p* = 0.028; OR = 2.03), with higher breastfeeding at discharge (47.2% vs. 30.6%, *p* = 0.020).

**Conclusion:**

Implementation of developmental care, including skin-to-skin contact, in preterm infants was associated with lower rates of late-onset sepsis, intraventricular hemorrhage, ROP, and postnatal growth failure, shorter hospital stays and mechanical ventilation, and higher rates of breastfeeding at discharge.

## Introduction

Advances in perinatal care have improved the survival of very and extremely preterm infants, yet the incidence of neonatal complications, postnatal growth failure, and preterm birth-related morbidities remains high and, in some cases, is even increasing (1, 2).

Preterm infants from the first days of life are exposed to multiple adverse factors in the neonatal intensive care unit (NICU), including light, noise, pain, and frequent invasive and non-invasive procedures, which negatively affect the immature organism and, together with severe clinical conditions, contribute to the development of both early and long-term complications. Early outcomes include infectious diseases, feeding difficulties, and respiratory disorders that tend to become chronic, while long-term outcomes involve impaired physical growth, neurodevelopmental, cognitive, and socio-emotional impairments, as well as visual and hearing deficits. The risk of developing complications increases with decreasing gestational age (3–7).

The fetal nervous system undergoes a highly active stage of development during the third trimester of pregnancy. Therefore, properly organized care is particularly important for preterm infants, as it helps minimize the negative impact of environmental factors and more closely replicate intrauterine conditions. Neurodevelopmental care, introduced by Professor Heidelise Als in 1986, involves a comprehensive set of medical and nursing interventions aimed at reducing the mismatch between extrauterine and intrauterine environments, decreasing stress in preterm infants in the neonatal intensive care unit, and promoting optimal neurobehavioral development (8).

It is now well established that the implementation of neurodevelopmental care in neonatal units, particularly in intensive care settings, reduces the risk of chronic lung disease and the need for prolonged oxygen support, improves sleep quality and self-regulation in preterm infants, and supports physiological homeostasis, including thermoregulation, respiratory and cardiovascular stability, as well as metabolic balance (9). Furthermore, developmental care has been associated with enhanced myelination, improved neurobehavioral and neurophysiological functioning, and better cognitive, motor, and language outcomes during long-term follow-up (10, 11).

Skin-to-skin contact is a fundamental component of neonatal developmental care, as it integrates and reinforces other aspects of care, creating the most supportive environment for the newborn, promoting physiological stability, facilitating active parental involvement, and supporting breastfeeding (12).

Despite growing evidence supporting the benefits of developmental care, its implementation in routine neonatal practice varies widely, particularly in resource-limited settings. While principles of developmental care are widely implemented in neonatal units in high-income countries (13, 14), in Ukraine only selected elements are beginning to be introduced. To date, evidence on the association between specific developmental care elements implemented in routine clinical practice and short-term clinical outcomes in preterm infants remains limited.

The original contribution of this study lies in the evaluation of developmental care elements implemented as part of routine neonatal practice rather than within a structured experimental program. Unlike many previous studies focusing on isolated interventions or highly controlled settings, this study assesses the association between a set of developmental care elements and multiple short-term clinical outcomes in extremely and very preterm infants in a real-world regional level III NICU.

The aim of this study was to evaluate the association between the implementation of developmental care elements, including skin-to-skin contact, and early clinical outcomes in preterm infants. Accordingly, the research question was whether these associations differed between extremely preterm and very preterm infants.

## Materials and methods

### Study design

This was a single-center, non-randomized observational before-after cohort study conducted in a regional level III perinatal center. The study compared outcomes of preterm infants treated before and after the implementation of developmental care as part of routine clinical practice, using a retrospective control group.

Short-term clinical outcome measures included the incidence of late-onset sepsis, bronchopulmonary dysplasia (BPD), intraventricular hemorrhage (IVH), periventricular leukomalacia (PVL), retinopathy of prematurity (ROP), feeding intolerance, duration of NICU stay, need for and duration of respiratory support, feeding type (breastfeeding vs. formula feeding), and growth parameters at discharge.

### Participant recruitment and group allocation

All preterm infants with a gestational age <34 weeks who required care in the NICU and met the inclusion criteria during the study periods were eligible for inclusion. Inclusion criteria: preterm infants with a gestational age (GA) below 34 weeks requiring care in the NICU and whose parents provided informed consent. Exclusion criteria: preterm neonates with congenital malformations, chromosomal abnormalities, or congenital infections, such as HIV, toxoplasmosis, rubella, cytomegalovirus, and herpes.

The developmental care (DC) group included preterm infants admitted after the implementation of developmental care elements in routine clinical practice and was recruited prospectively. The standard care (SC) group comprised preterm infants treated during the period preceding the implementation of developmental care; data for this group were collected retrospectively from medical records. Because this was an observational before-after study with multiple short-term outcomes, the sample size was based on feasibility within the study periods.

A total of 100 preterm infants were initially eligible for inclusion in the DC group, of whom 91 were included in the final analysis (5 did not meet the inclusion criteria and 4 died during the early or late neonatal period). The Developmental Care (DC) group included 19 (20.9%) extremely preterm infants (24–28 weeks of gestation; DC-1) and 72 (79.1%) very preterm infants (29–32 weeks of gestation; DC-2). In the Standard Care group, 119 infants were included retrospectively, and data were extracted from medical records. This group included 21 (17.6%) extremely preterm infants (SC-1) and 98 (82.4%) very preterm infants (SC-2).

### Developmental care protocol and standard care

Developmental care was implemented through a predefined set of standardized elements integrated into routine clinical practice and applied to all infants in the DC group. These elements included 24/7 parental access to the NICU, joint parent–infant stays in post-intensive care wards, protection of infant sleep, neonatal pain management, support for breastfeeding with the use of human milk fortifiers, environmental modifications to reduce visual and auditory stressors, infant positioning, and parental involvement in routine care. Daily pain assessment was performed, and non-pharmacological analgesia for invasive and stressful procedures included administration of a 30% glucose solution and non-nutritive sucking. Skin-to-skin contact was the core component of developmental care.

Standard care included caring for infants in incubators, with brief and intermittent parental visits to the NICU and without parental involvement in routine care. Procedures were performed according to standard protocols without systematic consideration of infant behavioral state or sleep–wake cycles, and formula feeding predominated.

### Human research statement

The local ethical committee (I. Horbachevsky Ternopil National Medical University) approved the study (protocol No. 73, April 3, 2023), and informed consent was obtained from all participants. Research was conducted in accordance with the World Medical Association's Helsinki Declaration.

Given the observational before-after design of the study and the inclusion of multiple short-term outcomes, a formal *a priori* sample size calculation was not performed, and the sample size was determined by feasibility within the predefined study periods. Consequently, the study may have had limited power to detect smaller differences in some outcomes.

### Statistics

All statistical analyses were performed using StatSoft STATISTICA Version 13 (Tulsa, OK). Normality of distribution was assessed using the Shapiro–Wilk test. Continuous variables are presented as median (Lq;Uq) or mean ± SD, as appropriate. Normally distributed variables were compared using the independent samples *t*-test, whereas non-normally distributed variables were compared using the Mann–Whitney *U* test. Categorical variables are presented as absolute numbers and percentages and were compared using Fisher's exact test. Odds ratios (OR) with corresponding 95% confidence intervals (CI) were calculated for selected dichotomous outcomes. A two-sided *p*-value < 0.05 was considered statistically significant.

## Results

### Obstetric and neonatal characteristics of the study groups

In the Developmental Care group, there were 55 boys (60.4%) and 36 girls (39.6%), while in the Standard Care group, there were 66 boys (55.5%) and 53 girls (44.5%). Sex distribution stratified by gestational age (extremely and very preterm infants) is presented in Table 1.

**Table 1:** Antenatal and intrapartum characteristics of infants in the study groups.

Parameter	Statistical measure	Extremely preterm infants (GA ≤ 28 weeks)		*p*	Very preterm infants (GA > 28 weeks)		*p*
		Group DC-1 (*n* = 19) (Developmental Care)	Group SC-1 (*n* = 21) (Standard Care)		Group DC-2 (*n* = 72) (Developmental Care)	Group SC-2 (*n* = 98) (Standard Care)	
Maternal factors							
Maternal age, years	Me [Lq; Uq]	31 [27.0; 34.0]	32.0 [27.0; 34.0]	0.725	28.0 [25.0; 32.0]	28.0 [24.0; 32.0]	0.644
Preeclampsia, hypertension	*n* (%)	4 (21.1)	0	0.042*	14 (19.4)	22 (22.5)	0.390
Placental dysfunction	*n* (%)	11 (57.9)	8 (38.1)	0.175	46 (63.9)	43 (43.9)	0.007*
Polyhydramnios	*n* (%)	2 (10.5)	3 (14.3)	0.549	15 (20.8)	18 (18.4)	0.416
Antepartum bleeding	*n* (%)	5 (26.3)	1 (4.8)	0.071	12 (16.7)	2 (2.0)	<0.001*
Infectious pathology	*n* (%)	4 (21.1)	2 (9.5)	0.282	9 (12.5)	24 (24.5)	0.038*
Maternal somatic comorbidities	*n* (%)	9 (47.4)	2 (9.5)	0.009*	32 (44.4)	19 (19.4)	<0.001*
Thyroid disease	*n* (%)	2 (10.5)	0	0.219	10 (13.9)	12 (12.2)	0.463
Anemia	*n* (%)	9 (47.4)	2 (9.2)	0.009*	34 (47.2)	34 (34.7)	0.068
Mode of delivery							
Vaginal delivery	*n* (%)	7 (36.8)	17 (80.9)	0.044*	20 (27.8)	58 (59.2)	<0.001*
Cesarean section	*n* (%)	12 (63.2)	4 (19.1)		52 (72.2)	40 (40.8)	
Anthropometric and gender characteristics of infants							
Birth weight. g	M ± SD	917.39 ± 207.14	1,033.81 ± 211.65	0.087	1,559.72 ± 81.42	1,571.63 ± 18.01	0.800
Birth weight percentile. %	Me [Lq; Uq]	58.0 [29.0; 73.0]	58.0 [43.0; 87.0]	0. 448	63.0 [40.5; 78.0]	69.0 [43.0; 86.0]	0.266
Small for gestational age	*n* (%)	1 (5.3)	0	0.287	3 (4.2)	7 (7.1)	0.416
Male infants Female infants	*n* (%)	13 (68.4) 6 (31.6)	14 (66.7) 7 (33.3)	0.587	42 (58.3) 30 (41.7)	52 (53.1) 46 (46.9)	0.299
Early neonatal characteristics							
Apgar score < 7 at 1st minute	*n* (%)	18 (94.7)	13 (61.9)	0.015*	41 (56.9)	30 (30.6)	<0.001*
Apgar score < 7 at 5th minute	*n* (%)	10 (52.6)	9 (42.9)	0.381	11 (15.3)	16 (16.3)	0.514
Need for primary resuscitation	*n* (%)	18 (94.7)	13 (61.9)	0.015*	41 (56.9)	30 (30.6)	<0.001*
Need for intubation during primary resuscitation	*n* (%)	9 (47.4)	7 (33.3)	0.280	2 (2.8)	9 (9.2)	0.083

GA, gestational age; Me [Lq; Uq], median [lower quartile; upper quartile]; M, mean; SD, standard deviation.

Continuous variables are presented as medians (Lq; Uq) or mean ± SD, as appropriate, and were compared using the Mann–Whitney *U* test or the independent samples *t*-test, depending on distribution; categorical variables are presented as numbers (percentages) and were compared using Fisher's exact test.

* A statistically significant result (*p* < 0.05).

Birth weights of extremely and very preterm infants did not differ significantly between the groups (*p* = 0.087 and *p* = 0.800, respectively). Regarding gestational age, in the Developmental Care and Standard Care groups, extremely preterm infants had a median gestational age of 27.0 [26.0–28.0] and 28.0 [27.0–28.0] weeks, respectively (*p* = 0.085), while very preterm infants had a median gestational age of 31.0 [30.0–31.0] and 30.0 [30.0–31.0] weeks, respectively (*p* = 0.510). Thus, the study groups, stratified by extremely and very preterm infants, were comparable in terms of GA and birth weight (Table 1).

Maternal and antenatal factors were generally comparable between groups. Maternal age did not differ significantly [28.0 [25.0–33.0] vs. 29.0 [25.0–32.0] years; *p* = 0.706]. Mothers in the Developmental Care group had fewer infectious complications but more frequently experienced antepartum hemorrhage, preeclampsia with hypertension, placental dysfunction, and somatic comorbidities. Other factors showed no significant differences between groups (Table 1).

An Apgar score <7 at 5 min was recorded in 52.63% vs. 42.85% of extremely preterm infants and in 15.28% vs. 16.33% of very preterm infants in the developmental care and standard care groups, respectively, with no significant differences between groups. Enteral feeding was initiated on day 1 of life in both groups.

### Early outcomes of preterm infants receiving developmental care elements

The main morbidity indicators and early outcomes of care in the study groups are presented in Table 2. Late-onset sepsis occurred significantly less frequently in both extremely and very preterm infants in the DC group compared with the SC group (42.1% vs. 76.20%, *p* = 0.049 and 11.11% vs. 22.4%, *p* = 0.041 for extremely and very preterm infants, respectively). Intraventricular hemorrhage was significantly less frequent in very preterm infants in the DC group compared with the SC group (13.9% vs. 30.6%, *p* = 0.008), and retinopathy of prematurity occurred less frequently in extremely preterm infants in the DC group than in the SC group (26.3% vs. 61.9%, *p* = 0.025).

**Table 2:** Neonatal morbidity and short-term outcomes in preterm infants in the study groups.

Parameter	Statistical measure	Extremely preterm infants (GA ≤ 28 weeks)			Very preterm infants (GA > 28 weeks)		
		Group DC-1 (*n* = 19) (Developmental Care)	Group SC-1 (*n* = 21) (Standard Care)	*p*	Group DC-2 (*n* = 72) (Developmental Care)	Group SC-2 (*n* = 98) (Standard Care)	*p*
Length of hospital stay, day	Me [Lq; Uq]	61.0 [53.0; 67.0]	71.0 [61.0; 88.0]	0.037*	31.0 [25.0; 36.0]	47.0 [34.0; 56.0]	<0.001*
Length of NICU stay, days	Me [Lq; Uq]	26.0 [19.0; 47.0]	17.0 [11.0; 35.0]	0.172	9.0 [5.0; 14.0]	6.0 [4.0; 12.0]	0.085
Surfactant replacement therapy	*n* (%)	18 (94.7)	13 (61.9)	0.009*	24 (35.3)	18 (18.4)	0.020*
Respiratory distress syndrome (RDS)	*n* (%)	19 (100.0)	14 (66.7)	0.056	61 (84.7)	19 (19.4)	<0.001*
Mechanical ventilation	[*n* (%)]	15 (78.9)	12 (57.1)	0.129	19 (26.4)	31 (31.6)	0.285
Duration of mechanical ventilation, days	Me [Lq; Uq]	21.0 [16.0; 34.0]	16.5 [9.0; 36.0]	0.662	7.0 [5.0; 10.0]	8.5 [7.0; 14.0]	0.045*
Bronchopulmonary dysplasia	[*n* (%)]	11 (57.9)	11 (52.4)	0.488	3 (4.2)	6 (6.1)	0.421
Early-onset sepsis	[*n* (%)]	8 (42.1)	5 (23.8)	0.185	13 (18.1)	33 (33.7)	0.017*
Late-onset sepsis	[*n* (%)]	8 (42.1)	16 (76.2)	0.049*	7 (9.72)	22 (22.4)	0.041*
Necrotizing enterocolitis	[*n* (%)]	2 (10.5)	1 (4.8)	0.461	17 (23.6)	21 (21.4)	0.438
Intraventricular hemorrhage	[*n* (%)]	11 (57.9)	14 (66.7)	0.403	10 (13.9)	30 (30.6)	0.008*
ROP, stage ≥3	[*n* (%)]	5 (26.3)	13 (61.9)	0.025	3 (4.2)	3 (3.1)	0.505
Daily weight gain, g	M ± SD	21.54 ± 4.96	18.31 ± 2.99	0.041*	22.85 ± 4.90	18.57 ± 4.87	<0.001*
Body weight percentile at discharge, %	Me [Lq; Uq]	22.0 [7.0; 52.0]	8.0 [4.0; 25.0]	0.045*	35.0 [20.0; 50.0]	14.5 [5.0; 35.0]	<0.001*
Postnatal growth failure at discharge	[*n* (%)]	4 (21.1)	12 (57.1)	0.048*	7 (9.7)	37 (37.8)	<0.001*
Breastfeeding at discharge	[*n* (%)]	4 (21.1)	3 (14.3)	0.441	34 (47.2)	30 (30.6)	0.020*

GA, gestational age; Me [Lq; Uq], median [lower quartile; upper quartile]; ROP, retinopathy of prematurity; NICU, neonatal intensive care unit.

Continuous variables are presented as medians (Lq; Uq) or mean ± SD, as appropriate, and were compared using the Mann–Whitney *U* test or the independent samples *t*-test, depending on distribution; categorical variables are presented as numbers (percentages) and were compared using Fisher's exact test.

* A statistically significant result (*p* < 0.05).

Use of developmental care elements was associated with a lower odds of late-onset sepsis, IVH, and severe ROP in preterm infants. These associations, stratified by gestational age, are presented in Table 3.

**Table 3:** Odds ratios for selected clinical outcomes associated with the implementation of developmental care elements.

Outcome	Gestational age group	OR (95% CI)
Late-onset sepsis	Extremely preterm	0.23 (0.06–0.88)
Late-onset sepsis	Very preterm	0.37 (0.15–0.93)
IVH	Very preterm	0.37 (0.16–0.81)
ROP stage III	Extremely preterm	0.22 (0.06–0.85)
Postnatal growth failure	Extremely preterm	0.20 (0.05–0.81)
Postnatal growth failure	Very preterm	0.18 (0.07–0.43)
Prolonged breastfeeding	Very preterm	2.03 (1.08–3.81)

OR (95% CI), odds ratio and corresponding 95% confidence interval.

IVH, intraventricular hemorrhage; ROP, retinopathy of prematurity.

The length of hospital stay was significantly shorter in both age subgroups of the DC group compared with the SC group (*p* = 0.037 and *p* < 0.001 for extremely and very preterm infants, respectively). The need for mechanical ventilation did not differ between groups, although its duration was shorter in the DC-2 group compared with SC-2 (*p* = 0.045). The incidence of BPD did not differ between the groups.

Postnatal growth failure (birth weight <10th percentile at discharge) was significantly less frequent in the DC group, occurring in 26.3% of extremely preterm and 9.7% of very preterm infants, compared with 57.1% and 37.8% in the SC group (*p* = 0.048 and *p* = 0.061, respectively). Daily weight gain was significantly higher in both age subgroups of the DC group (21.54 ± 4.96 g vs. 18.31 ± 2.99 g; *p* = 0.041 in extremely preterm infants, and 22.85 ± 4.90 g vs. 18.57 ± 4.87 g; *p* < 0.001 in very preterm infants), Table 2. Implementation of developmental care strategies was associated with a lower odds of postnatal growth failure in extremely and very preterm infants (Table 3).

The rate of breastfeeding at discharge was higher in the DC-2 group compared with the SC-2 group (47.2% vs. 30.6%, *p* = 0.020). Developmental care, including skin-to-skin contact, was associated with a higher likelihood of prolonged breastfeeding in very preterm infants (Table 3).

## Discussion

Prior research has examined perinatal factors affecting early and long-term outcomes in preterm infants, but increasing evidence shows that environmental conditions and the quality of early care are equally important for the development and maturation of the immature nervous system (8). Our findings suggest that early care practices in the NICU are linked to variations in early neonatal outcomes. In this study, the use of developmental care elements, particularly skin-to-skin contact, was associated with lower rates of late-onset sepsis, IVH, ROP, postnatal growth failure, and shorter hospital stays. These findings suggest that developmental care may represent a potentially modifiable factor in early neonatal outcomes among preterm infants.

Baseline differences between the study groups should be carefully considered when interpreting the results. Several maternal and perinatal risk factors, including maternal comorbidities, obstetric complications, and indicators of early neonatal instability, were more prevalent in the developmental care group. These factors are known to be associated with increased neonatal morbidity and could potentially confound comparisons of outcomes between groups. Notably, despite these baseline imbalances, the developmental care group showed more favorable clinical outcomes. Nevertheless, given the non-randomized observational before-after design, residual confounding cannot be excluded, and the findings should be interpreted accordingly.

Late-onset sepsis occurred significantly less frequently in preterm infants receiving developmental care or its components compared with those managed with standard care across both gestational age groups. Nosocomial infections continue to be a major challenge in extremely and very preterm infants requiring prolonged NICU treatment (15), highlighting the critical importance of early interventions and optimized care practices to reduce infection risk. Specifically, our study confirms that developmental care was associated with a 4.4-fold and 2.7-fold lower odds of late-onset sepsis in extremely preterm infants and very preterm infants respectively. It is well established that a fundamental component of developmental care, skin-to-skin contact, is associated with positive clinical outcomes, including a reduced risk of hypothermia and nosocomial infections, as well as lower rates of other complications, particularly among extremely and very preterm infants (16).

Our results align with evidence from the Cochrane Database of Systematic Reviews, showing that Kangaroo Mother Care (KMC) with skin-to-skin contact significantly reduces the risk of nosocomial infections and sepsis in preterm infants (17). Additional studies have reported fewer sepsis episodes and, when infections occurred, less severe courses in infants receiving KMC compared with standard care (18).

Skin-to-skin contact reduces the risk of infections by enhancing the skin barrier function of preterm infants and maintaining optimal hydration. Adequate skin hydration protects against harmful substances and excessive inflammatory responses, supporting normal skin function (19). Furthermore, elements of developmental care, including skin-to-skin contact, proper positioning, swaddling, and non-nutritive sucking, enhance comfort and physiological stability, reducing pain and stress in neonates (20, 21). This decreases sympathetic nervous system activity, lowers corticosteroid secretion, and reduces the risk of stress-induced infections. Skin-to-skin contact also promotes oxytocin release, increases melatonin levels, and concurrently lowers cortisol, thereby normalizing hormonal balance in infants in the NICU A prospective study of 150 preterm infants (GA ≤ 36 weeks) showed a significant reduction in salivary cortisol after skin-to-skin contact, particularly in infants with lower gestational age (22). Taken together, these findings suggest that reduced stress exposure and improved physiological stability associated with developmental care, particularly skin-to-skin contact, may contribute to lowering NICU-related stress and indirectly reducing the risk of late-onset infections in preterm infants.

Our study showed that IVH was significantly less frequent in infants receiving developmental care compared with those in the standard care group. In addition to well-recognized physiological risk factors for IVH in preterm infants (low antenatal steroid use, transfer to higher-level hospitals, respiratory and cardiovascular instability, with associated hypoxia, acidemia, and cerebral blood flow fluctuations), the quality of NICU care plays a crucial role (23). Implementation of developmental care strategies was associated with 2.5-fold lower odds of IVH in very preterm infants, suggesting a potential protective association with the neurodevelopmental interventions. These interventions may enhance autonomic stability and reduce cerebral vulnerability, thereby contributing to neuroprotection (24, 25).

Our findings are consistent with previous studies showing that the Newborn Individualized Developmental Care and Assessment Program (NIDCAP), a structured neurodevelopmental care program, is associated with a lower incidence and severity of IVH and reduced ventricular dilatation (26). Notably, routine neonatal care is linked to most episodes of hypoxia, whereas skin-to-skin contact improves oxygen saturation, blood pressure stability, and cerebral oxygenation (27). Overall, the results indicate that developmentally supportive care may mitigate stress-related cerebral instability and hypoxic events, thereby potentially reducing the risk of IVH in very preterm infants.

Our study showed a significantly shorter length of hospital stay in infants receiving developmental care compared with standard care. These results are consistent with evidence from systematic reviews and meta-analyses demonstrating that Family-Centered Care and Kangaroo Mother Care reduce hospitalization duration and improve outcomes in preterm infants (28, 29). The Alberta integrated family-centered care model in neonatal intensive care units also reduced the length of hospital stay in preterm infants, without concomitant increases in readmissions or emergency room visits (30). This supports the value of implementing developmental and family-centered care practices in neonatal intensive care units.

Comparative analysis of developmental and standard care showed that infants in the developmental care group required significantly shorter durations of mechanical ventilation. These findings align with McAnulty et al., who reported shorter durations of mechanical ventilation and oxygen therapy in infants receiving NIDCAP care vs. standard care (31). A recent study by Klein et al. found that the implementation of Infant- and Family-Centered Developmental Care strategies was associated with significantly shorter mechanical ventilation, earlier extubation, and improved short-term respiratory outcomes in extremely preterm infants compared with standard care in the NICU (32). Although data directly quantifying the painful and stressful nature of invasive mechanical ventilation itself are limited, this intervention is accompanied by a high number of potentially painful and stressful procedures, including intubation, reintubation, frequent endotracheal suctioning, and skin injury related to adhesive devices (33). Repeated exposure to such procedures may contribute to prolonged stress and pain in preterm infants and has been associated with adverse outcomes (34). Overall, shorter mechanical ventilation among infants receiving developmental care may be associated with lower cumulative stress and potentially more favorable short-term outcomes.

In addition to improvements in respiratory support and length of hospital stay, our study found a significant reduction in the incidence and severity of retinopathy of prematurity in infants receiving developmentally supportive care compared with those managed with standard care. Using elements of developmental care was associated with lower odds of stage III ROP in extremely preterm infants by 2.2 times. These findings are consistent with literature describing the neuroprotective and vasoprotective effects of developmental interventions. Components of developmental care, such as skin-to-skin contact and environmental modifications, enhance physiological stability and reduce the duration of oxygen therapy, thereby limiting oxygen-related oxidative stress contributing to ROP development (35, 36). Effective stress and pain management further contribute to ROP prevention by reducing stress hormone levels, as prolonged cortisol release promotes pro-inflammatory cytokine production and disrupts normal retinal vascular development (37). By minimizing oxygen exposure and stress-related inflammatory responses, developmental care may attenuate key pathogenic pathways of ROP. Additionally, the lower rates of sepsis and intraventricular hemorrhage and shorter durations of mechanical ventilation observed in the developmental care group suggest that developmental care strategies may indirectly reduce the incidence and severity of ROP in preterm infants (38). Thus, developmentally supportive care may influence multiple interrelated mechanisms, including oxygen exposure, inflammation, and stress regulation, and thereby potentially reduce the incidence and severity of ROP in preterm infants.

Our results suggest that developmental care was associated with more favorable physical growth parameters and supports breastfeeding in preterm infants. Infants receiving developmental care had significantly higher daily weight gain and lower rates of postnatal growth failure compared with those receiving standard care, with developmental care was linked to approximately fivefold lower odds of postnatal growth failure in extremely preterm and by 5.5-fold in very preterm infants. Our findings are consistent with those of Wang et al., who reported that Kangaroo Mother Care in neonatal intensive care units is associated with improved physical growth and breastfeeding outcomes. Specifically, infants receiving daily skin-to-skin contact showed better anthropometric progress and greater feeding efficiency compared with those receiving standard care, highlighting skin-to-skin contact as a key component of developmental care (39). O'Brien et al. also reported that Family Integrated Care in NICUs is associated with improved weight gain compared with standard care, reflected by more favorable weight *Z*-score changes on day 21 of life (14). Similar results have been observed in comparisons between infants cared for under the NIDCAP program and those receiving standard care (31). A recent systematic review including 10 randomized controlled trials involving 1,153 neonates reported that both short- and long-duration skin-to-skin contact effectively improve growth rates and breastfeeding outcomes in low-birth-weight infants (40).

The main factors contributing to postnatal growth restriction in preterm infants are related to early feeding challenges, the severity of the infant's condition, comorbidities, inadequate coverage of metabolic needs, high energy expenditure, as well as physiological postnatal weight loss and fluid imbalance (41). In contrast, developmental care with skin-to-skin contact has been shown to promote physical growth, enhance lactation, and increase the rate of exclusive breastfeeding at discharge. Skin-to-skin contact stabilizes body temperature, reducing energy expenditure and allowing resources to be directed toward tissue growth (17, 42). Contact with the mother stimulates lactation and breastfeeding, ensuring adequate nutrient intake (43). By reducing stress and pain, developmental care decreases activation of the sympathetic nervous system and the hypothalamic-pituitary-adrenal axis, lowering cortisol and catecholamine levels, stabilizing cardiovascular and respiratory functions, and improving sleep (20, 44, 45). This also preserves metabolic efficiency, optimizes gastrointestinal perfusion and nutrient absorption, and promotes growth hormone and oxytocin release, collectively creating optimal conditions for growth and neuroendocrine development (46).

Alongside improved physical growth, our study showed that among very preterm infants, a higher proportion in the developmental care group were breastfed at discharge, and developmental care doubled likelihood that they would continue breastfeeding. A key factor in this process is Kangaroo Mother Care with skin-to-skin contact, which, by stimulating oxytocin release, enhances lactation and supports the establishment of early and prolonged breastfeeding in preterm infants (20). Studies have shown that preterm infants receiving Kangaroo Mother Care experienced breastfeeding for a longer duration and had higher rates of exclusive breastfeeding than those receiving standard care (47). Oras et al. found that the duration of skin-to-skin contact during hospitalization significantly affects the time to achieve full breastfeeding (48). These observations suggest that skin-to-skin contact as part of developmental care may facilitate the initiation and maintenance of breastfeeding in preterm infants by supporting maternal-infant bonding and lactation physiology (49).

## Strengths and limitations

The strengths of this study include its conduct in a single perinatal center with consistent clinical protocols and treatment approaches, which reduces variability between centers. Comparing outcomes from the period of standard care prior to the implementation of neurodevelopmental care elements with current practice allowed us to assess the real-life impact these care modifications.

Given the non-randomized observational before-after design and baseline differences between groups, the findings should be interpreted as associations rather than evidence of causal relationships. In addition, residual confounding from unmeasured clinical or organizational factors cannot be excluded. Secular changes in NICU practice over time, improvements in clinical protocols and staff experience may also have influenced outcomes independently of the developmental care intervention. Finally, long-term neurodevelopmental outcomes were not assessed, highlighting the need for future prospective studies.

## Conclusion

Implementation of developmental care strategies, including skin-to-skin contact, in preterm infants was associated with differences in early clinical outcomes. Infants receiving developmental care had lower rates of late-onset sepsis, intraventricular hemorrhage, and retinopathy of prematurity, as well as shorter durations of mechanical ventilation and hospital stay, improved postnatal growth, and higher rates of breastfeeding at discharge compared with those receiving standard care.

These findings suggest that early, developmentally supportive care may represent a feasible strategy in the NICU and may be associated with more favorable clinical and growth-related outcomes in preterm infants. Even partial implementation of developmental care elements was linked to measurable benefits, underscoring the importance of integrating structured developmental care practices into routine neonatal care. However, further prospective studies are needed to evaluate long-term neurodevelopmental outcomes and the effects of fully implemented developmental care programs.

## Data Availability

The raw data supporting the conclusions of this article will be made available by the authors, without undue reservation.
